# Tendon and Ligament Genetics: How Do They Contribute to Disease and Injury? A Narrative Review

**DOI:** 10.3390/life12050663

**Published:** 2022-04-29

**Authors:** William J. Ribbans, Alison V. September, Malcolm Collins

**Affiliations:** 1 School of Health, The University of Northampton, Northampton NN1 5PH, UK; 2 The County Clinic, Northampton NN1 5DB, UK; 3 Division of Physiological Sciences, Department of Human Biology, Health Sciences Faculty, University of Cape Town, Cape Town 7700, South Africa; alison.september@uct.ac.za (A.V.S.); malcolm.collins@uct.ac.za (M.C.); 4 Health Through Physical Activity, Lifestyle and Sport Research Centre (HPALS), Department of Human Biology, Health Sciences Faculty, University of Cape Town, Cape Town 7700, South Africa; 5 International Federation of Sports Medicine (FIMS), Collaborative Centre of Sports Medicine, Department of Human Biology, University of Cape Town, Cape Town 7700, South Africa

**Keywords:** genetics, epigenetics, tendon injury, ligament injury, tendinopathy

## Abstract

A significant proportion of patients requiring musculoskeletal management present with tendon and ligament pathology. Our understanding of the intrinsic and extrinsic mechanisms that lead to such disabilities is increasing. However, the complexity underpinning these interactive multifactorial elements is still not fully characterised. Evidence highlighting the genetic components, either reducing or increasing susceptibility to injury, is increasing. This review examines the present understanding of the role genetic variations contribute to tendon and ligament injury risk. It examines the different elements of tendon and ligament structure and considers our knowledge of genetic influence on form, function, ability to withstand load, and undertake repair or regeneration. The role of epigenetic factors in modifying gene expression in these structures is also explored. It considers the challenges to interpreting present knowledge, the requirements, and likely pathways for future research, and whether such information has reached the point of clinical utility.

## 1. Introduction

It has been estimated that tendon and ligament injuries account for 30–50% of all sporting injuries [1]. The impact on people’s overall well-being, ability to work, and participate in exercise is significant. The health economic burden of managing such conditions is huge [2,3]. Is the prevalence of such injuries simply related to increased societal involvement in sport increasing exposure to extrinsic risk factors, such as running or other high impact activities? Alternatively, has the more sedentary lifestyle of many, combined with increasing levels of obesity, simply led to deconditioning and increased loading causing failure of these critical structures? How much does our inherited genotype influence our risk profile? This review considers the impact of genetic factors on the development of tendon and ligament injury and disease. What is our present state of knowledge of tendon and ligament structure and function? What do we understand about the influence of genetic factors and how might this knowledge be used in the future to reduce risk?

## 2. The Injury Causation Model and Jar Model

**Figure 1:**
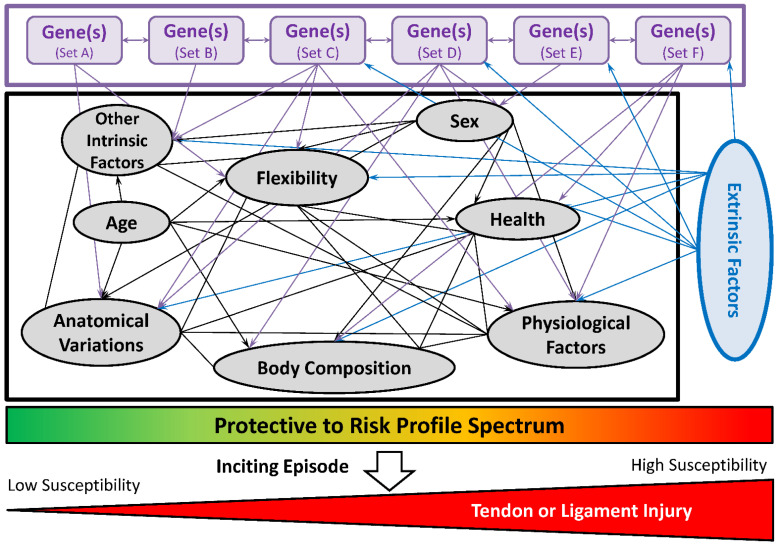
A hypothetical diagram showing the complex interaction of numerous genetic factors and extrinsic factors in determining an individual’s specific profile along the ‘reduced to increased risk (predisposed) spectrum’.

Tendon and ligament pathologies usually follow the injury causation model described by Meeuwisse in 1994 [4] and refined by Bittencourt in 2016 [5]. A complex interaction of intrinsic (including genetic factors) and extrinsic factors influences an individual’s specific profile along the ‘reduced to increased risk (predisposed) spectrum’. Following an inciting episode, such as an injurious event, the problem becomes symptomatic to the patient (Figure 1). An injury may manifest itself as an acute episode or chronic condition. For the latter, there is often initial damage that is asymptomatic. Further subsequent inciting episodes, which can be identified by the patient, causes the damage to become symptomatic [6].

**Figure 2:**
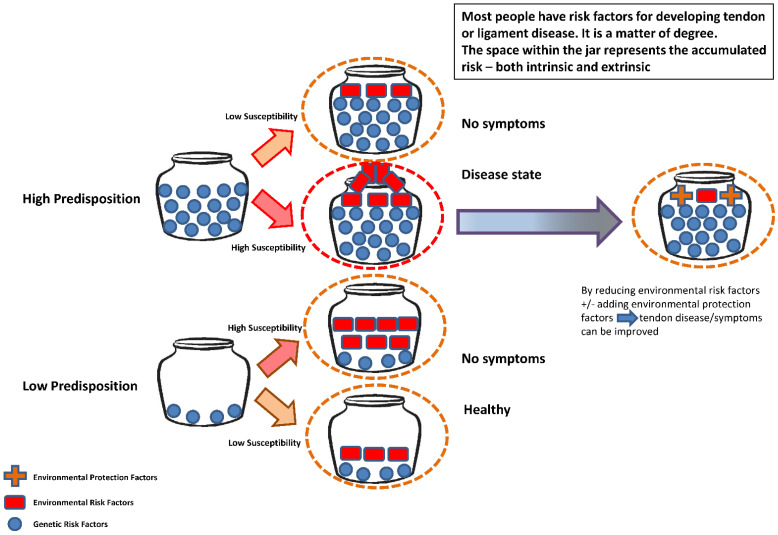
The Jar Model. Accumulative risk factors for developing chronic tendon and ligament disease. Adapted from the ‘Jar Model’ of Jehannine Austin used in psychiatric genetic counselling (Austin, 2008) [7].

People inherit genetic factors which can result in an increased or reduced risk for many potential tendon and ligament injuries. However, the possession of such factors does not necessarily lead to the clinical development of such multifactorial conditions. Austin’s Jar Model for genetic risk was developed for use in psychiatric counselling [7] and applied to other areas of medicine, including cancers. It can be used in discussing risk for developing musculoskeletal damage (Figure 2).

Extrinsic factors can be overwhelming, leading to injury such as in a tackle-playing sport causing ligament injury. However, for many instances of damage (usually sub-acute or chronic), both genetic and non-genetic intrinsic elements combine with extrinsic or environmental factors. Once the jar has ‘overflowed’, the damage becomes apparent. Careful review of modifiable risk factors (such as flexibility and body composition variables) combined with attempts to reduce their influence and the adoption of protective factors (such as improved nutrition and rest intervals) can improve tendon and ligament health. Medications such as steroids and certain fluoroquinolone antibiotics (examples include ciprofloxacin), can initiate tendon damage that can be ameliorated following their cessation [8,9].

## 3. Genetic Involvement in Musculoskeletal Condition

Most intrinsic risk factors for common tendon and ligament injuries have a genetic contribution [10]. For instance, flexibility has a heritability component estimated to between 64 and 70% [11,12]. Familial studies have reported a 40% heritability between twins for lateral epicondylitis [13], a five times increased risk for rotator cuff issues in siblings [14], and twin studies describing a knee anterior cruciate ligament (ACL) tear risk heritability of 69% [15].

Some rare orthopaedic conditions, such as pseudoachondroplasia and osteogenesis imperfecta, are caused by gene mutations [16,17]. However, most sports injuries are caused by extrinsic factors (such as training load) interacting with an individual’s genetic background and other intrinsic factors [18]. Additionally, some medical conditions with a genetic background increase the risk of tendon pathology, including various seropositive and seronegative rheumatological conditions (such as gout and ankylosing spondylitis), Ehlers–Danlos syndrome, and other endocrine and metabolic disorders [19].

We note a spectrum of genetic-related connective tissue disorders. At one extreme are conditions associated with a classical Mendelian inheritance pattern—such as osteogenesis imperfecta [20], Ehlers–Danlos syndrome [21] and Marfan’s syndrome [22]. Establishing the genetic pattern of these conditions usually involves techniques such as linkage analysis or direct gene sequencing [23]. At the other end of the spectrum are conditions with a multifactorial aetiology often involving multiple and complex interactions between various genes with non-genetic intrinsic and extrinsic factors. These include gene–gene and gene with non-genetic factor interactions, making the investigation of the genetic contribution to such conditions more complicated.

## 4. Types of Genetic Studies

Depending on the prevalence of the phenotype and, therefore, the sample size, several study designs can be applied to investigate genetic influences on the development of multifactorial phenotypes such as tendon and ligament injuries.

### 4.1. Family Studies

The most basic of these formats are family studies. Disease inheritance patterns between family members indicate the degree of hereditability of such conditions. Examples of autosomal dominant (e.g., Huntington’s disease), autosomal recessive (e.g., phenylketonuria), and sex-linked related (e.g., Vitamin D resistant rickets with hypophosphatemia) are readily identified. However, the interactions of many factors (including genetic and non-genetic factors), such as a shared environmental load, influence the susceptibility to complex multifactorial clinical conditions such as tendon and ligament injuries. Therefore, identifying several members of a family with an identical injury is not common and, for this reason, a classical inheritance pattern is usually not identifiable. Twin studies represent a unique opportunity to identify the shared heritability component between twins together with a potential shared environmental exposure.

### 4.2. Case-Control Studies

Case-control genetic association studies allow interrogation and comparison of large data sets both within a population and between populations of different geographical locations and ancestry. This has been a popular method of identifying genetic risk factors of common multifactorial phenotypes including tendon and ligament pathologies [24]. However, it requires rigour in the phenotyping of cases and controls. Cases should be well defined, and diagnoses confirmed using preferably ‘gold standard’ methodology, such as imaging or surgical confirmation. Additionally, the multifactorial nature of the susceptibility, a comprehensive medical history, sporting history (including training regimes), medicines’ use, and familial injury history should be recorded for both cases and controls.

Controls’ selection is equally important, and individuals should be matched for sex, age, body mass index (BMI), and sports participation exposure and level, and any other potential confounders. Both the cases and controls may harbour genetic and non-genetic elements, which may confer an increased or decreased risk to sustaining a potential tendon or ligament injury. It is the balance of these multifactorial risk factors which will determine if the injury presents.

### 4.3. Hypothesis-Free Approach

Almost all past and current research has focused on the candidate gene approach where knowledge of a given gene and the injury is assumed (Section 4.2 above). There has been progress towards a hypothesis-free approach using the application of next generation sequencing technologies, such as genome wide association studies (GWAS). Most GWAS studies have used canine models [25,26,27] although one human study highlighted three independent DNA sequence variants associated with ACL rupture—albeit of borderline significance [28]. A whole exome sequencing (WES) approach was also recently undertaken in a twin family study, where 11 novel variants were highlighted for further exploration in ACL injury susceptibility [29]. Recently, Gibbons used a hybrid approach of WES on targeted participants and applied a tiered filtering strategy to identify potentially biologically relevant new candidate-variants within previously implicated genes. This allowed further prioritisation in larger independent cohorts [30]. Currently, there are no whole genome sequencing datasets specific to exercise-related injury phenotypes, such as tendon and ligament injuries. In the future, genetic susceptibility would gain from research characterising the genome in relation to tendon and ligament injury.

## 5. Tendon: Structure, Function and Genetic Research

Tendons transfer the forces generated within muscles to their bone insertions. Tendons are composed of a heterogeneous population of tendon cells embedded within an extracellular matrix (ECM) consisting of collagen fibres, elastin fibres, proteoglycans, glycosaminoglycans, and glycoproteins.

### 5.1. Tendon Cells

Ninety to 95% of the cellular population within the mature tendon, which synthesise and regulate the components of the ECM, consist predominately of tenocytes and immature tenoblasts [31]. Tenocytes are predominately found within the fascicles between the collagen fibres and have an intricate network of connections using processes producing intercellular links via gap junctions [32]. The more rounded and metabolically active tenoblasts are primarily situated between fascicle units within the inter-fascicular matrix (IFM) [33,34]. Tendon cell activity alters with exposure to normal stresses, injury, and ageing. One to 4% of the cells within tendons are tendon stem/progenitor cells (TSPCs) which have similar characteristics to mesenchymal stem cells [35].

### 5.2. Collagen

**Figure 3:**
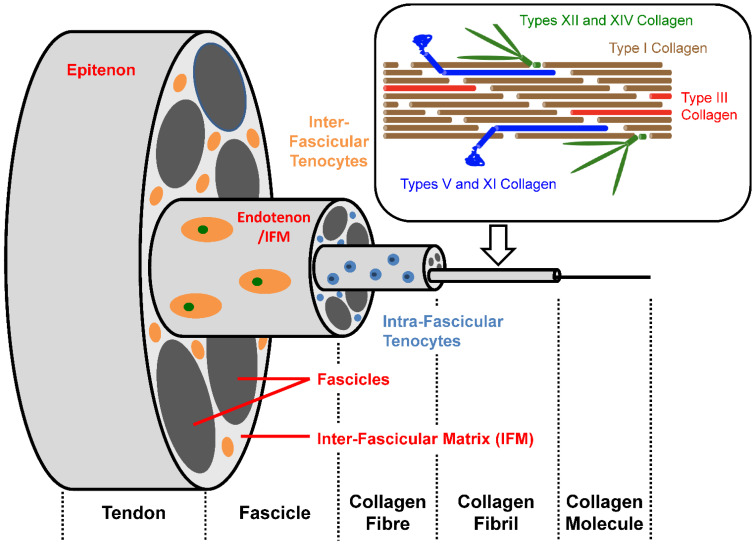
Hierarchical structure of the tendon.

Within the ECM, collagen is the major structural protein and constitutes 60–85% of the dry tendon weight. The collagen is arranged in a hierarchical manner [36] (Figure 3).

#### 5.2.1. Type I Collagen

Type I collagen accounts for up to 90% of total collagen content. Type I collagen fibrils are the tendon’s primary structural elements. They provide tensile strength enhanced by cross-linking. Fibrils aggregate to fibres and, once again, fibres combine to form fascicles. Each fascicle is surrounded by an endotenon or IFM. Fascicles combine to form the tendon entity, which is bound together by a surrounding epitenon. A paratenon surrounds many tendons, such as the Achilles (Table 1).

Two studies, a South African cohort of Achilles tendon disease and a Turkish cohort of lateral epicondylitis of the elbow, investigated the *COL1A1* rs1800012 functional DNA sequence variant with tendon disease and neither found an association [47,48]. However, later sub-analysis of acute Achilles tendon rupture in the South African cohort found that possession of a relatively rare TT genotype protects against injury [49]. More recently Gibbon [50] explored the *COL1A1* rs1007946-rs1800012 haplotype and implicated G-T to be associated with reduced risk of Achilles tendinopathy and rupture (Table 2). Other collagen types, although in a minority in terms of mass, play an important part in determining collagen configuration, strength, and optimal function.

#### 5.2.2. Type III Collagen

Type III collagen, encoded by *COL3A1*, is the second most common type comprising up to 10% of total collagen (Table 1). To date, no studies have investigated the association of *COL3A1* variants with tendon injuries (although they have been undertaken in ligament injuries—see Section 6.1.3).

#### 5.2.3. Type V Collagen

Type V collagen performs a similar role to Type III collagen. *COL5A1* encodes for its α1 chain and is found close to the ABO gene on chromosome 9q34 (Table 1). A South African cohort study described four variants within the 3’-untranslated region (3’-UTR), which were independently associated with the development of chronic Achilles’ tendinopathy (AT). Additionally, a protective genotype for a single variant was identified [51]. The variants located within the 3’-UTR appear to influence the stability of the mRNA. This region appears to play an important role in post-transcriptional regulation [52,53]. The authors hypothesise that the C and T alleles of rs12722 influences *COL5A1* mRNA stability and possibly through alternate mRNA structural forms, which may affect type V collagen expression. They further hypothesise that the mRNA stability is enhanced with the functional form associated with increased chronic AT, leading to increased type V collagen [54]. Collins further postulated on how increased type V collagen content was associated with altered mechanical properties of tendons and increased injury susceptibility [55]. A later study reported other genotypes, also within the functional *COL5A1* 3’-UTR, in South African and Australian populations that showed increased risk for chronic AT [56]. More recently, RNA sequencing analyses in torn rotator cuff tissue vs. control tissue, showed that *COL5A1* gene expression was markedly (3.01×) increased in tears, demonstrating its role in healing and/or remodelling [57].

The effect of altered genotypes in the *MIR608* gene, which encodes a small micro-RNA (miRNA), was undertaken in Australian and South African groups [58]. An increased risk of AT was demonstrated with the rs4919510 CC genotype. This miRNA can bind to a recognition sequence within the *COL5A1* and other genes 3’-UTR and inhibits translation. It was the first non-coding gene to be associated with soft-tissue injuries and increased understanding of the complex mechanisms involved in the regulation of type V collagen production. A GWAS study investigating a large cohort, including samples of different ancestry, suggested that *MIR608* rs4919510 showed moderate evidence for AT susceptibility—although not at the level of significance required for a GWAS study [29] (Table 2).

**Table 2:** Summary of genetic research and tendon injuries: Collagen types I and V.

Collagen Type	Reference	Country	Variant	Pathology	Subjects	Controls	Comments
Type I	[46]	South Africa	*COL1A1* rs1800012	Achilles’ tendinopathy and rupture	126	125	No significant association
	[47]	Turkey	*COL1A1* rs1800012	Lateral epicondylitis (common extensor origin)	183	123	No significant association
	[58]	Spain	*COL1A1* rs1800012	Patellar tendon injury	15	0	No difference between severity of injury and gene variants. No controls were included
	[48]	South Africa and Sweden (controls only)	*COL1A1* rs1800012	Achilles’ tendon rupture	41	581	Significant association of the TT genotype protecting against acute Achilles’ tendon rupture
	[49]	South Africa and UK	COL1A1 rs1107946 rs1800012	Achilles’ tendinopathy and rupture	216	193	Significant association of the (G-T) haplotype protecting against Achilles tendinopathy and rupture
Type V	[50]	South Africa	COL5A1 rs12722	Achilles’ tendinopathy and rupture	111	129	Significant protective association with variant in tendon disease
	[59]	Italy	COL5A1 rs12722	Bilateral quadriceps rupture	9	0	No controls but association with a polymorphism and tendon rupture
	[60]	Italy	COL5A1 rs12722	Bilateral quadriceps rupture	1	0	No controls but association with a polymorphism and tendon rupture (same finding as Galasso)
	[58]	Spain	COL5A1 rs12722	Patellar tendon injury	15	0	No difference between severity of injury and gene variants. No controls were included
	[55]	South Africa, Australia	COL5A1 rs12722 COL5A1 rs3196378	Achilles’ tendinopathy and rupture	178	342	Significant protective association with variant of *COL5A1* rs12722 in tendon disease In Australian cohort only, rs31996378 associated with tendinopathy
	[57]	South Africa, Australia	COL5A1 3’-UTR rs71746744, rs1134170, rs16399 MIR608 rs4919510	Achilles’ tendinopathy and rupture	160	342	All variants within the untranslated region of the *COL5A1* gene and MIR variant were associated with Achilles tendon disease

Green indicates an association and red indicates no association found.

#### 5.2.4. Type XI Collagen

Type XI collagen is found in many structures including articular cartilage, bone, and muscle (Table 1). Variants in *COL11A1* and *COL11A2* interact with one another and with a *COL5A1* 3’-UTR variant to modulate the AT risk in South African and Australian cohorts [43] (Table 3).

#### 5.2.5. Types XII, XIV, and XXVII Collagens

None of the investigated functional variants of *COL12A1* or *COL14A1* which encode the α-chains of types XII and XIV collagen have been associated with AT [59]. However, *COL12A1* may be associated with acute Achilles tendon ruptures [59]. Saunders investigated several *COL27A1* gene variants that encode Type XXVII collagen but could not identify any independent association with AT [60] (Table 1 and Table 3).

### 5.3. Proteoglycans

Proteoglycans (PGs) represent up to 5% of tendon dry weight. They consist of a core protein attached to one or more glycosaminoglycans (GAGs). The GAG’s negative charge binds water, which makes up 55–70% of the total tendon weight. PGs are found between the ECM’s collagenous structural components (Table 4). There are no reported genetic studies associated with tendon PG. However, ligament PG genetic variants have been investigated (see Section 6.2.2).

### 5.4. Glycoproteins

Glycoproteins are a large family of structurally and functionally diverse proteins to which a carbohydrate group(s) is covalently attached. Two important tendinous glycoproteins are tenascin-C and cartilage oligomeric matrix protein (COMP) (Table 4).

**Table 3:** Summary of genetic research and tendon injuries: Collagen types XI, XII, XIV, and XXVII.

Collagen Types	Reference	Country	Variant	Pathology	Subjects	Controls	Comments
Type XI	[43]	South Africa, Australia	*COL11A1* rs1676486, rs3753841 *COL11A2* rs1799907	Achilles’ tendinopathy and rupture	184	338	No significant independent associations found Genes encoding structural and functionally related type XI (*COL11A1* and *COL11A2*) and type V (*COL5A1*) collagens interact with one another to collectively modulate Achilles’ tendinopathy risk
Type XII	[59]	South Africa, Australia	*COL12A1* rs240736, rs970547	Achilles’ tendinopathy and rupture	137	131	No significant associations found There may be an association with Achilles tendon rupture
Type XIV	[59]	South Africa, Australia	*COL14A1* rs4870723, rs1563392	Achilles’ tendinopathy and rupture	137	131	No significant associations found
Type XXVII	[60]	South Africa, Australia	*COL27A1* (several polymorphisms)	Achilles’ tendinopathy and rupture	178	340	No significant associations found among variants

Green indicates an association and red indicates no association found.

**Table 4:** Regulatory and structural components of tendons.

Component	Form and Types	Functions	Associated Diseases
Proteoglycans (PGs)	PGs Found between extra-cellular matrix’s (ECM) collagenous structural components PGs include Decorin, Lubricin, and Versican Consist of a core protein attached to one or more glycosaminoglycans (GAGs)	Decorin (80%): roles include collagen fibrillogenesis and potentially enhancing strength by facilitating load transfer between discontinuous collagen fibrils. Most abundant in tendinous areas most subject to tension Lubricin: Found close to tendon periphery and aids tendon gliding with lubrication Versican: Found within the interfascicular matrix (IFM)	
Glycoproteins	Tenascin-C (TNC) Protein is a hexamere, which binds to both ECM components and tenocyte surface receptors Produced by *TNC* gene located close to the ABO gene on chromosome 9q34 *TNC* gene expression appears influenced by tension loading [61]	Important role in regulating the tenocytes need to interact with ECM components Involved in cell adhesion and signalling, affecting cell proliferation and migration [62,63,64] Appears to increase in the presence of tendon pathology [65]	Mutations within *TNX* gene (a tenascin family member) causes an autosomal recessive form of EDS from tenascin-X deficiency [66]
	Thrombospondins (THBS) Family of glycoproteins performing dynamic role within ECM Cartilage oligomeric matrix protein (COMP) Also known as thrombospondin V Most abundant tendinous glycoprotein Links to type I collagen and has five subunits allowing linkage between multiple fibrils Found within the inter-fibrillar matrix [67] and tissues subject to significant loading, such as tendons, ligaments, cartilage, menisci, and intervertebral discs Thrombospondin 2 (*THBS2*) Mediates cell-to-cell and cell-to-matrix interactions Involved in cell-to-cell adhesion and ECM communication Thrombospondin type 1 domain–containing protein 7A This protein regulates focal adhesions during angiogenic cell migration	Role debated COMP-null knock out mice did not demonstrate any tendon abnormality [68] Others reported in horses that COMP levels during growth corelate with mechanical strength at skeletal maturity [66]	Pseudo-achondroplasia is caused by a heterozygous mutation in the gene encoding COMP [16] A *COMP* gene mutation can cause multiple epiphyseal dysplasia (MED)
Elastin and Microfibrils	Elastin (ELN)	Elastin provides elasticity to tendons, allowing them to stretch and return to their original state. Plays important load-bearing role in tendons and ligaments.	*ELN* rs2071307 variant has been associated with other multifactorial ECM conditions, such as aortic stenosis [69] and aortic aneurysm [70] *ELN* rs2071307 and *FBN2* rs331079 variants associate with aortic and intracranial aneurysms, respectively, causing ECM disruption Fibrillin-1 abnormalities have been associated with Marfan’s syndrome and Fibrillin-2 have with Beal’s syndrome or congenital contractural arachnoldactyly [71,72]
	Microfibrils, such as the glycoprotein, Fibrillin: Found in ECM and become incorporated into insoluble microfibrils	Plays a role in early elastogenesis acting as a scaffold for elastin deposition [73]	

#### 5.4.1. Tenascin-C

Tenascin-C (*TNC*) plays an important role in regulating tenocytes that need to interact with ECM components (Table 4). The first research establishing a genetic association for a predisposition to Achilles tendon pathology was published in 2005 and implicated *TNC* [74]. A South African case-control association study reported that a Guanine-Thymine Dinucleotide repeat variant was associated with Achilles tendon injuries. A second study investigating different *TNC* variants (rs13321; rs2104772; rs1330363) found altered frequencies between cases and controls in Australian and South African populations—but did not reach statistical significance [75] (Table 5). However, the study did implicate a genetic region spanning both the *TNC* and the *COL27A1* genes using haplotype analysis. More recently, the application of WES analyses assisted the identification of potential functional *TNC* variants implicating the *TNC* gene, specifically rs1061494 and the T-T haplotype (rs1061494-rs2104772) with increased risk of Achilles tendinopathy in a South African cohort [30]. This study therefore provided evidence that the risk susceptibility to Achilles’ tendinopathy is most likely within the *TNC* gene rather than within the *COL27A1* gene locus.

Several *TNC* variants have been associated with rotator cuff injury [76,77]. Previously implicated *TNC* variants (rs1138545, rs72758637, rs7021589) have been replicated in a GWAS study using the UK biobank to be associated with an increased risk of rotator cuff injury [57,76]. Tashjian reported increased gene expression, for *TNC* (2.2×) in tears vs. controls [57] (Table 5).

#### 5.4.2. COMP and Other Thrombospondins

COMP, also known as thrombospondin 5, is the most abundant tendinous glycoprotein. Thrombospondin 2 (*THBS2*) mediates cell-to-cell and cell-to-matrix interactions and is involved in cell-to-cell adhesion and ECM communication (Table 4). *COMP* (rs730079; rs28494505) and *THBS2* (rs9505888; rs6422747) variants failed to show significant difference in Achilles tendon studies in Australian and South African cohorts [75]. No human genetic studies exist relating to the potential role of Thrombospondin 2 in tendon pathology, although its absence has been associated with connective tissue abnormalities in mice [76].

A GWAS study identified rs575224171 within the gene *THSD7A* encoding the endothelia protein thrombospondin type 1 domain, containing protein 7A, to be associated with increased risk of rotator cuff injury [57]. These authors hypothesised that gene variants may lead to poor rotator cuff angiogenesis, predisposing individuals towards increased tear risk. RNA sequencing analyses established a 2.6× decreased expression of this gene within rotator cuff tears vs. control tissues [57].

### 5.5. Elastin and Microfibrils

Elastin represents 1–10% of a tendon’s dry weight and is found in both the IFM and within the fascicles—especially around tenocytes. Microfibrils, such as the glycoprotein Fibrillin, are found in the ECM and become incorporated into insoluble microfibrils (Table 4). South African, Australian, and British cohorts did not find an association between the *ELN* rs2071307 variant and risk of developing Achilles’ tendon pathology [78,79]. However, the *FBN2* rs331079 variant was associated with risk for Achilles’ tendon disease and ACL ruptures [78].

**Table 5:** Summary of genetic research and tendon injuries: Glycoproteins in the ECM and elastin and fibrillin.

Type	Protein	Reference	Country	Variant	Pathology	Subjects	Controls	Comments
Glycoproteins in the ECM	Tenascin-C (TNC)	[74]	South Africa, Australia	*TNC*—GT dinucleotide repeats	Achilles’ tendinopathy and rupture	144	127	Association with the number of GT repeat polymorphism and the risk of tendinopathy and rupture
		[60]	South Africa, Australia	*TNC* rs13321, rs2104772, rs1330363	Achilles’ tendinopathy and rupture	179	339	No significant associations found among variants
		[80]	Spain	*TNC* rs2104772	Patellar tendon injury	15	0	No difference between severity of injury and gene variants. No controls were included
	Cartilage oligomeric matrix protein (COMP)	[75]	South Africa, Australia	*COMP* rs730079, rs2849450 *THBS2* rs9505888	Achilles’ tendinopathy and rupture	178	340	No significant associations found among variants
Elastin and Fibrillin	Elastin (ELN) and Fibrillin (FBN)	[78]	South Africa, Australia	*ELN* rs2071307 FBN2 rs331079	Achilles’ tendinopathy and rupture	135	239	No association with the ELN variant and tendon pathology Significant association between the FBN2 gene variant and tendon disease
		[81]	UK	*ELN* rs2071307	Achilles’ tendinopathy and rupture	108	131	No association with the ELN variant and tendon pathology
		[80]	Spain	*ELN* rs2289630	Patellar tendon injury	15	0	No difference between severity of injury and gene variants. No controls were included

Green indicates an association and red indicates no association found.

### 5.6. Tendon Development, Homeostasis, and Remodelling

Tendon ECM homeostasis and remodelling are maintained by complex enzyme systems. These include matrix metalloproteases (MMPs), ADAMTSs (a disintegrin and metalloproteinase with thrombospondin motifs) and ADAMs (a disintegrin and metalloproteinase), tissue inhibitors of MMPs (TIMPs), and growth factors like the transforming growth factor-ß (TGF-ß) families [82] (Table 6). Genetic research undertaken in this area of tendon development, homeostasis, and remodelling is summarised in Table 7.

#### 5.6.1. MMPs, TIMPs, ADAMTSs, and ADAMs

The balance between MMPs and TIMPs is necessary to maintain tendon homeostasis and remodelling [82]. If intrinsic control of these systems is compromised by extraneous factors, the tendon’s ability to respond appropriately to loading will be affected, risking tendon disease (Table 6). No association was reported of *ADAMTS2*, *ADAMTS5*, *ADAMTS14*, and *ADAM12* variants with Achilles’ tendon pathology in South African and Australian cohorts [83]. However, Raleigh found significant associations for *MMP3* variants and AT (but not rupture) in a South African population [84]. In a British population, El Khoury found no associations in Achilles’ pathology groups overall—although subgroups did show some correlations [83]. Furthermore, El Khoury found a significant association with a variant within the *TIMP2* gene rs478932 for Achilles’ tendon pathology in both a South African and Australian cohort. Different genotypes were overrepresented in the subject groups in each population [83]. Gibbon explored the *MMP3* locus in an Australian cohort with AT and identified a 6A-G-C-G haplotype (rs3025058, rs679620, rs591058, rs650108) with reduced risk [85].

**Table 6:** Tendon development, homeostasis and remodelling.

Families	Functions	Key Regulators	Associated Diseases
Matrix metalloproteases (MMPs) (23 family members)	Four subgroups: Collagenases Gelatinases Stromelysins Membrane-type Broad proteolytic activity against collagen and other ECM compounds	MMP3: degrades different collagen types, proteoglycans, and fibronectin, and laminin	Fluoroquinolone antibiotics, which are associated with increased Achilles rupture risk [86], can affect MMP expression in human tendons [9,87]
A disintegrin and metalloproteinase with thrombospondin motifs (ADAMTSs) (20 family members)	Procollagen processing ECM remodelling	ADAMTS2 and 14: regulate conversion of procollagen to collagen ADAMTS5: cleaves proteoglycans, e.g., aggrecan	ADAMTS2 gene mutation causes EDS dermatosparaxis type
A disintegrin and metalloproteinase (ADAMs) (19 family members)	Proteases	ADAM12: a marker of skeletal muscle regeneration and binds insulin growth factor binding protein-3 (IGFBP-3)	
Tissue inhibitors of metalloproteases (TIMPs)	Inhibit MMPs activities Balance between MMPs and TIMPs is necessary to maintain tendon homeostasis and remodelling		
Transforming growth factor beta (TGF-ß) superfamily	ECM homeostasis and remodelling	TGF-ß1 and growth differentiation factor 5 (GDF-5), can increase Achilles tendon strength in animals [88]. Mechanical loading releases TGFß-1 and aids cell growth, proliferation, differentiation, and migration, as well as cell death (apoptosis). Involved in collagen and proteoglycan synthesis. GDF-5 (also called CDMP-1 (cartilage-derived morphogenetic protein 1) or BMP-14 (bone morphogenic protein 14). Expressed in developing CNS and maintenance, development and repair of bones, cartilage, and various other musculoskeletal soft tissues, including tendons.	*GDF-5* gene mutations (CDMP1) implicated in Hunter–Thompson type dwarfism and in Grebe Syndrome (characterized by short stature, extra digits, and short and deformed extremities) [89,90,91,92].
Bone morphogenetic proteins (BMPs) (20 family members)	Grouped into subfamilies of the TGF-ß superfamily Growth factors and cytokines in many tissues in the body		
Fibroblast growth factors (FGFs) (22 family members)	Influence cell development and maturation by binding to receptors (FGFR) triggering intracellular events		Point mutation in FGFR3 can lead to achondroplasia.

#### 5.6.2. Transforming Growth Factor-ß (TGF-ß) Superfamily

The TGF-ß superfamily has a similar role as the MMP/TIMP system in ECM homeostasis and remodelling (Table 6). Mechanotransduction is the process of converting mechanical forces into a cellular response. Tendon exposure to increased loading (within safe physiological limits) causes tenocytes to increase collagen synthesis and enhance tendon load resistance. It is the end goal of sensible incremental training [92]. The reverse occurs with inactivity. If a tendon’s tensile load is temporarily decreased, there is a reduction in secreted ECM structures including Type I collagen and COMP [93]. TGF-ß appears to be a major regulator of tendon development secondary to mechanical loading [94]. The exact mechanism of how loading activates the TGF-ß signalling pathway appears to involve an induction of scleraxis (*Scx*) and other markers, such as tenomodulin [95]. This promotes the synthesis and secretion into the ECM of collagen and other ECM components. *TGF-ß* and *GDF-5* genes functional variants have been studied with an association established for *GDF-5* rs143383, but not *TGF-ß* rs1800469, with risk of Achilles’ tendinopathy [96].

#### 5.6.3. Bone Morphogenic Glycoproteins (BMP)

BMPs are grouped into subfamilies of the TGF-ß superfamily and function as growth factors or cytokines. Originally studied for their effect upon bone and cartilage formation, they are recognised to have a widespread function as signallers and regulators of many organs systems’ development [35]. A significant association was reported in a Brazilian mixed-injury cohort for the *BMP4* variant (rs2761884) in tendinopathies [97].

#### 5.6.4. Fibroblast Growth Factors (FGFs)

FGFs are required for normal development and cell maturation. They bind to receptors (FGFR), triggering intracellular events. Salles studied a group of Brazilian volleyball players with variously located tendinopathies. However, none of the investigated *FGF3*, *FGF10*, and *FGFR1* variants were associated with altered risk for tendinopathy [97]. Similarly, no associations of the same genes with rotator cuff tears in American patients were reported [98]. Conversely, Motta found significant associations of the *FGF3*, *FGF10*, and *FGFR1* variants with rotator cuff disease in large Brazilian case-control study [99]. 

**Table 7:** Summary of genetic research and tendon injuries: Development, homeostasis and remodelling.

Families	Reference	Country	Variant	Pathology	Subjects	Controls	Comments
A disintegrin and metalloproteinase with thrombospondin motifs (ADAMTSs) A disintegrin and metalloproteinase (ADAMs)	[83]	South Africa, Australia	*ADAMTS2* rs1054480 *ADAMTS5* rs226794 *ADAMTS14* rs4747096 *ADAM12* rs4747096	Achilles’ tendinopathy and rupture	173	248	No significant association with Achilles’ disease ADAMTS14 associated with a later onset of disease
Matrix metalloproteases (MMPs) and Tissue inhibitors of metalloproteases (TIMPs)	[84]	South African	*MMP3* rs591058, rs650108, rs591058	Achilles’ tendinopathy and rupture	114	98	Variants significantly associated with chronic tendinopathy No increased risk of rupture with variants Lowest risk of tendinopathy associated with combination of a MMP3 rs679620 and COL5A1 rs12722 variants
	[81]	UK	MMP3 rs679620 *TIMP2* rs4789932	Achilles’ tendinopathy and rupture	118	131	MMP3 variant significantly associated with tendinopathy and rupture groups in males but not females or overall. TIMP2 variants overrepresented in tendinopathy group
	[83]	South Africa, Australia	*TIMP2* rs4789932	Achilles’ tendinopathy and rupture	173	248	SNP significantly overrepresented in tendinopathy group
	[85]	Australia	*MMP3* rs679620, rs591058, rs650108, rs3025058	Achilles’ tendinopathy	79	195	An association was found with a 6A-G-C-G haplotype (rs3025058, rs679620, rs591058, rs650108) with reduced risk for Achilles tendinopathy
Transforming Growth Factor-ß Superfamily (TGFß) Growth/differentiation factors (GDF)	[96]	South Africa, Australia	*TGFB1* rs1800469 *GDF5* rs143383	Achilles’ tendinopathy and rupture	171	238	No associations with TGFB1 genotype variant GDF5 variant associated with Achilles’ disease.
Bone morphogenetic proteins (BMPs)	[97]	Brazil	*BMP4* rs2761884	Mixed—Achilles; Patellar; Rotator cuff; Hip abductors	52	86	Significant association with BMP4 variant for tendinopathy
Fibroblast Growth Factors	[97]	Brazil	*FGF3* rs7932320, rs1893047, rs12574452, rs4631909, rs4980700 *FGF10* rs1448037 rs900379, rs1011814, rs593307 FGFR1 rs13317	Mixed—Achilles; Patellar; Rotator cuff; Hip abductors	52	86	No significant associations with tendinopathy
	[98]	USA	*FGF3* *FGF10* *FGFR1*	Rotator Cuff tears	175	2595	No significant associations
	[99]	Brazil	*FGF3* rs12574452 FGF10 rs11750845, rs1011814 *FGFR1* rs13317	Rotator Cuff tears	203	207	All showed significant associations with rotator cuff tears

Green indicates an association and red indicates no association found.

### 5.7. Cell Death (Apoptosis) and Inflammation in Tendons

Apoptosis is a natural phenomenon in many living tissues. In tendons, damaged tenocytes’ removal is facilitated by cytokine activity. Excessive tendon loading can increase apoptosis and affects the cell population’s abilities to respond effectively to exercise with secondary effects upon the ECM leading to tendon disease [100]. The role of inflammation in chronic tendinopathy has long been debated [101]. Early research utilising microscopic examination of tendinopathic tissue and biochemical analysis reported no evidence of the normal elements associated with ‘classical’ inflammation in chronic tendon injuries [102,103,104,105]. Animal work indicated that early tendinosis was associated with tenocyte stimulation rather than apoptosis and modulated by growth factors such as insulin-like growth factor 1 (IGF-1) [106]. However, the authors were unable to comment on the chronic effect of prolonged loading on cell survival. (Table 8).

#### 5.7.1. Interleukins

Inflammatory pathways throughout the body involve numerous elements, interacting in a complex manner and resulting in gene expression alterations, apoptosis, and detrimental changes to the ECM. The protein family of interleukins are intimately involved in the inflammatory pathway. Interleukins are upregulated in early tendinopathy and involved in the inflammatory cascade and remodelling activities [114]. September reported interleukin gene-gene interactions with *COL5A1* rs12772, suggesting that type V collagen may be regulated by certain inflammatory mediator proteins in the IL-1β-signalling pathway [115]. Altering the amount of type V collagen expression could impact α1(V) collagen chains and, thereby, the collagen tendon fibril diameter and ultimately tendon capacity (Table 9).

#### 5.7.2. Caspases

Caspases are a family of protease enzymes that are integral to programmed cell death (apoptosis). The South African research group reported that two *CASP8* genotypes had significant associations with AT [116] (Table 9).

#### 5.7.3. Nitric Oxide Synthase (NOS) Enzymes

Nell found no association with gene variants for *NOS2* and *NOS3* and AT [115]. However, Brookes reported a reduced risk for AT with the NOS2 rs2779249 heterozygote variant, but no association with *NOS2* rs2248814 [117] (Table 9).

### 5.8. Angiogenesis

Angiogenesis is the formation of new blood vessels from the existing vasculature. Tendons and ligaments have a poor blood supply and low metabolic rate. Consequently, their healing capacity is low [118]. Histopathological examination of chronic AT specimens shows marked increases in angiogenesis [110]. It is hypothesized that this is triggered by mechanical loading and designed to promote tendon remodelling. Further studies have identified increased levels of pro-angiogenic expression profiles and, specifically, vascular endothelial growth factor A after tenocyte mechanical loading [119,120,121]. Poorly regulated angiogenesis may lead to distortion of the neat parallel collagen fibril array in the tendon ECM. Increased levels of angiogenic associated proteins have been noted in both ruptured tendons and ligaments and including degenerative tendons [121,122,123]. Angiogenesis elements are placed centrally within the network of partners regulating key ECM components within tendon and ligament.

Several functional variants within the *VEGFA* (rs699947, rs1570360, and rs2010963) gene have been explored and a risk haplotype was implicated both in a (i) South African cohort and a (ii) combined South African and British cohort of mid portion chronic AT [124]. Specifically, the *VEGFA* A-G-G (rs699947 C/A–rs1570360 G/A–rs2010963 G/C) inferred haplotype was associated with increased risk of AT. This haplotype includes the collective alleles associated with decreased *VEGFA* gene transcription and a corresponding lower VEGFA plasma level [125]. Therefore, it is reasonable to hypothesise that these allele combinations would potentially contribute to limiting the capacity of the structure to regulate ECM remodelling within a hypovascular tendon [122]. The authors did not report an association with Achilles’ tendon risk for any of the variants explored in *KDR* (rs2071559 and rs1870377) [123] (Table 8). 

Like other gene loci, differences in associations at the *VEGFA* and *KDR* loci have been noted in populations of different ancestry. One study reported no associations in two KDR polymorphisms (rs1870377; rs2071559) with AT in South African and UK cohorts [124] (Table 9).

### 5.9. Other Areas of Genetic Study in Tendons

#### 5.9.1. ESRRB

Estrogen-related receptor beta (ERR-β) is a nuclear receptor encoded by *ESRRB* (Estrogen Related Receptor Beta) gene. Its function is unknown; however, a similar protein in mice plays an essential role in placental development. It appears to influence the expression of PPARGC1 and ESRR-inducing regulator muscle 1 (PERM1) in skeletal muscle. Motta identified two 2 SNPs in the *ESRRB* gene that were associated with rotator cuff disease [99]. Teerlink found a significant association for rotator cuff injury with an *ESRRP* rs17583842 variant [98] (Table 9).

#### 5.9.2. Defensin ß1

Defensins form a family of microbicidal and cytotoxic peptides made by neutrophils. Defensin ß1 is encoded by the *DFNB* gene. It resists microbial organisms from attaching to epithelial surfaces. Motta found the *DEFB-1* rs1800972 SNP to be associated with a preventive effect for rotator cuff tears [99] while Teerlink found no association with *DEFB*-1 [98] (Table 9).

**Table 9:** Summary of genetic research and tendon injuries: Inflammatory cascade, apoptosis, and others.

Type	Protein	Reference	Country	Variant	Pathology	Subjects	Controls	Comments
Inflammatory Cascade and Apoptosis	Interleukins (ILs)	[116]	South Africa, Australia	*IL1B* rs1143627, rs16944 *IL1RN* rs2234663 *IL6* rs1800795	Achilles tendinopathy and rupture	175	369	No independent associations. In combination with COL5A1 rs12722 these alleles had significant association with Achilles’ tendinopathy
	Caspases (CASP)	[115]	South Africa, Australia	*CASP8* rs3834129, rs1045485	Achilles tendinopathy and rupture	166	358	Significant association with both polymorphisms
	Nitric Oxide Synthases (NOS)	[115]	South Africa, Australia	*NOS2* rs2779249 *NOS3* rs1799983	Achilles tendinopathy and rupture	166	358	No significant associations
		[117]	UK	*iNOS* rs2779249 iNOS rs2248814	Achilles tendinopathy and rupture	132	145	Significant association (protective effect) with rs2779249 No association with rs2248814
Angiogenesis	Angiogenic Factors	[124]	South Africa and UK	*VEGFA rs699947, rs1570360, rs2010963* *KDR rs1870377*, *rs2071559*	Achilles tendinopathy	120 130	108 87	Significant association with all 3 VEGFA polymorphisms No association with the KDR polymorphisms
Others	Defensins (DEF)	[98]	USA	*DEFB1*	Rotator Cuff tears	175	2595	No association
		[99]	Brazil	*DEFB1* rs1800972	Rotator Cuff tears	203	207	Significant association (protective effect)
	Estrogen-related receptor ß (ESRRB)	[98]	USA	*ESRRB* rs17583842	Rotator Cuff tears	175	2595	Significantly associated with rotator cuff disease
		[99]	Brazil	*ESRRB* rs1676303, rs4903399	Rotator Cuff tears	203	207	Significantly associated with rotator cuff disease

Green indicates an association and red indicates no association found.

### 5.10. Ageing in Tendons

Diseased and damaged tendons increase with age. For instance, 11–37% develop tears in an ankle peroneal tendon during their lifetime [126]. Long exposure to mechanical stresses and the increasing inefficiency of tendon repair are implicated. It is a classic example of the Injury Causation model (Figure 1). As a result, performance and function are impaired [127]. Many age-related changes occur to tendons during their lifetime and differences are apparent in the way men and women respond to tendon loading [128]. Additional knowledge has been derived from animal research, especially equine studies and it has been proposed that the ability of pluripotent mesenchymal stem cells to differentiate into tenocytes reduces with time. Tendon stem cells become less numerous with age and their ability to differentiate and produce competent mature tenocytes reduces [129]. The senescence-inhibited gene within the tenocyte is downregulated [130]. The ageing tenocyte’s complement of produced proteins (proteome) is restricted [131]. The proteins affected include those with roles involved in cell survival and death, cytoskeletal changes, and antioxidant response.

The ability to repair damaged tendons is also affected by non-collagenous ECM protein turnover, including cytokines and various growth factors, and a disruption to the fine homeostatic mechanisms outlined in Section 5.6 [132]. Equine research has revealed that with age, the tendons’ protein turnover diminishes [133], glycosaminoglycans increase [134], the type III collagen increases proportionately [38], and collagen fibril diameter diminishes [135]. Changes occur to the IFM with reduced protein turnover and elasticity, increasing the risk of injury [136,137]. MMP activity reduces tendon strength [127]. There is additional evidence that age carries with it a reduced capacity to resolve inflammation [138]. In addition, degenerative human tendons can have altered responses to reactive oxygen species with age, and therefore oxidative stress may be an important pathway in tendinopathy development [139].

The progressive alteration in tendon function involves a complex interplay between many influences. This includes not only our inherited genetic material but temporal changes to gene expression regulation, including an array of epigenetic mechanisms [140]. The potential role of epigenetics will be elaborated upon in Section 9.

## 6. Ligament: Structure, Function, and Genetic Research

Ligaments span joints attaching at either end to bones. Like tendons, they are fibrous, dense connective tissues. They are designed to resist excessive load, control joint motion, prevent instability, and have a vital proprioceptive role on account of their rich innervation. They have a similar hierarchical structure to tendons. However, the degree of packing of the collagen is slightly different. Whereas tendons organise collagen fibres in an orderly, parallel orientation, ligament organisation is more random and less parallel. This allows ligaments to respond to tensile loads in different directions. The cellular elements tend to be more randomly distributed and rounder in shape than tendons. There is a higher percentage of proteoglycans and water and reduced percentage of collagen. The elastin content in ligament is higher than tendons.

### 6.1. Collagen

#### 6.1.1. Type I Collagen

There has been considerable interest in the genetic architecture of the *COL1A* gene and susceptibility to tendon and ligament injuries. Differences in the genetic susceptibility to acute and chronic injuries have been linked to rs1107946 (−1997 G/T) and rs1800012 (+1245 G/T), within the *COL1A1* gene. The rare TT genotype of the Sp1 binding site variant (rs1800012) was associated with decreased risk for acute injuries such as shoulder dislocations [141], ACL ruptures [47], and acute soft tissue ruptures [49]. A meta-analysis reported the association of the rs1800012 TT genotype with reduced risk for sports-related tendon and ligament injuries [142]. The same genotype has been associated with increased risk for intervertebral disc degeneration in the elderly and increased risk for lumbar disc disease in young military recruits [143,144]. The alternate rs1800012 GG genotype was reported to reduce risk for ACL ruptures sustained while skiing [145]. The rs1107946 variant, which is in linkage disequilibrium with rs1800012, has been independently associated with the risk of skiing-associated ACL ruptures [146]. Haplotype analyses with these two functional variants have been associated with ACL rupture risk in a Polish cohort [147]. The current theory proposes that these functional promotor variants work in concert to regulate *COL1A1* expression [148].

However, several studies have failed to reflect an association between these *COL1A1* variants and susceptibility to several musculoskeletal soft tissue injury phenotypes. This may result from insufficient power of the studies to detect the rare rs1800012 TT genotype in and may explain conflicting results when comparing data from larger combined analyses to that of smaller independent cohorts [50]. No associations were noted in the Chinese Yunnan Han ACL samples for the *COL1A1* or *COL5A1* locus [149]. However, the TT genotype of the *COL1A1* Sp1 binding site polymorphism has been reported to be significantly underrepresented in South African participants with ACL ruptures [150] (Table 10).

#### 6.1.2. Type V Collagen

As in AT research, variants within the 3’-UTR of the *COL5A1* gene have been associated with ACL rupture susceptibility, specifically in females [152,153,154], and more recently with ligament injuries [155]. Laguette explored the intron 4-exon 5 region of *COL5A1*, which was previously implicated with ligament injuries in a canine model but found no significant associations in a South African ACL cohort [156]. The *COL5A1* rs12722 C/T and *COL5A1 rs13945* C/T polymorphisms were also associated with reduced ACL injury risk in male skiers [157]. Furthermore, Suijkerbuijk reported an association with ACL ruptures and *COL5A1* rs12722 in a combined Swedish and South African cohort [158] (Table 11).

#### 6.1.3. Types III and XII Collagen

Associations have been noted for variants in *COL3A1* [151,152], and *COL12A1* [149,159] with ACL rupture risk, while others reported no significant associations [147,161].

### 6.2. Regulatory and Structural Components of the Extracellular Matrix

#### 6.2.1. Tenascin-C

Historically, there has been much interest in the associations between the *TNC* gene and its neighbouring genes with tendon and ligament injury susceptibility. Gibbon explored this region for ACL susceptibility. Variants within the *TNC* gene: rs2104772 and a TT haplotype (rs1061494 and rs2104772) were associated with ACL susceptibility using a tailored WES and bioinformatics approach [30]. However, no associations for the *TNC* locus were noted in a Polish cohort [162]. More functional research is required to understand the biological significance underpinning this genetic locus and tendon and ligament injury susceptibility.

#### 6.2.2. Proteoglycans

Recent studies have investigated proteoglycans. Variants within their controlling genes have been implicated with susceptibility to ACL ruptures in independent cohorts from South Africa [163,164] and Poland [165] (Table 12).

#### 6.2.3. MMPs

As in tendinopathy, the *MMP* locus (chr11q22) has been association with susceptibility to ACL ruptures. Posthumus demonstrated that *MMP3* rs679620 variant may interact with several other MMP loci, *MMP10* rs485055, *MMP1* rs1799750, and *MMP12* rs2276109, to collectively contribute to ACL rupture susceptibility in a South African cohort [167]. The *MMP3* rs3025058 variant, which is tagged by rs679620, was independently associated with ACL ruptures in a Thai population [168]. No associations were noted when *MMP1* rs1799750, *MMP10* rs486055, and *MMP12* rs2276109 variants were explored with ACL rupture susceptibility in a Polish cohort [169]. The *MMP* genes have been investigated with several different exercise-related phenotypes and conflicting associations have been noted. This suggest that there may be specific genetic signatures which are inherited together and underpin specific exercise-related phenotypes, which still require functional unravelling (Table 13).

#### 6.2.4. Transforming Growth Factor Superfamily

Variants in several such genes controlling the TGF superfamily have been explored with an association to ACL rupture risk. These include variants within the *TGF-β receptor III* (TGFβR3) and the *TGF-β induced* (TGFβI) genes. An independent association of *TGFBR3* rs1805113 G allele with a decreased risk of ACL injury has been described. Additionally, a genetic interval between *TGFBR3* rs1805113-rs1805117 was associated with ACL injury risk in a South African cohort [158].

GDF5 plays a critical role in tendon and ligament repair. Variant analyses within *GDF5* gene have shown conflicting risk associations with ACL injury and larger studies are required to understand the significance of this locus with ACL injury risk [170,171].

### 6.3. Signalling Factors

#### 6.3.1. Interleukins

Investigation of interleukins have shown similar findings to tendinopathy. An inferred allele combination (*IL1B*, *IL6*, *IL6R*, and *COL5A1*) was associated with ACL rupture risk [158,172,173]. Differences were noted at the alleles implicated for the *IL1RN* rs2234663 and *IL6* rs1800795 loci. The functional consequence of these genetic loci was subsequently explored [157]. Cells treated with either hrIL-β or hrTNF-α expressed altered levels of *BGN* mRNA (which encodes for the biglycan PG) and *COL5A1* mRNA depending on their *IL1B*-high risk or *IL1B*-low genotype profiles. Evidence suggests that the inflammatory micro-environment together with an individual’s genetic profile can modulate ECM expression of tendon and ligament components and thereby potentially impact these structures’ functional capacity.

#### 6.3.2. Caspases

Both Rahim and Seale have reported associations between caspase functional gene variants and ACL ruptures [174,175].

**Table 13:** Summary of genetic research and ligament injuries: Development, homeostasis and remodelling and inflammatory cascade and apoptosis.

Type	Protein	Reference	Country	Variant	Pathology	Subjects	Controls	Comments
Development, Homeostasis, and Remodelling	TGFβ Growth Factor Superfamily	[156]	South Africa	TGFB1 rs1442, TGFBR3 rs1805113, rs1805117	ACL rupture	249	210	Significant association
		[170]	South Africa	*GDF5* rs1413383	ACL rupture	126	214	No significant association
		[171]	China	*GDF5* rs1413383	ACL rupture	286	500	Significant association
	Matrix metalloproteases (MMPs)	[167]	South Africa	*MMP1* rs1799750 *MMP3* rs679620 *MMP10* rs486055 MMP12 rs2276109	ACL rupture	129	216	Significant association
		[168]	Thailand	*MMP3* rs3025058, rs679620	ACL rupture	86	100	Significant association
		[169]	Poland	*MMP1* rs1799750 *MMP10* rs486055 *MMP12* rs2276109	ACL rupture	228	202	Significant association
Inflammatory Cascade, and Apoptosis	Interleukins (ILs)	[173]	Poland	IL1B rs1143627, rs16944 *IL6R rs2228145, IL6* rs1800795	ACL rupture	229	194	Significant association
		[158]	South Africa and Sweden	IL1B rs16944, *IL6* rs1800795 *IL6R* rs2228145	ACL rupture	79	116	Significant association
		[176]	South Africa	IL1B rs16944, *IL6* rs1800795 *IL6R* rs2228145	ACL rupture	234	232	Significant association
	Caspases (CASP)	[172]	South Africa	*CASP8* rs3834129	ACL rupture	234	232	Significant association
		[175]	South Africa	*CASP8* rs383412, rs1045485, rs13113	ACL rupture	102	116	Significant association

Green indicates an association and red indicates no association found.

#### 6.3.3. Angiogenesis

Independent and haplotype associations were noted for *VEGFA* functional variants (rs699947, rs1570360, and rs2010963) with ACL rupture susceptibility [172,174,177,178] including contrasting associations between ACL rupture and AT susceptibility. For example, the *VEGFA* rs699947 CC was associated with increased risk of non-contact ACL ruptures [174] but associated with a reduced risk of AT [124]. Similarly, the inferred haplotype, associated with increased VEGF production [125], was more often observed with an increased risk of ACL rupture whereas the low-*VEGF* producing haplotype was associated with a reduced risk of injury [174]. In contrast, the low-*VEGF* producing haplotype was associated with increased risk of tendinopathy [124]. Following a pathway-based approach, including DNA variants within the interleukin and the angiogenesis encoding genes, Rahim highlighted that *VEGFA* rs699947 CC, *VEGFA* rs2010963 GC, BMI, and age remain significant biological components in ACL rupture susceptibility [176].

Evidence suggests that a lower-level blood flow increase after running is associated with higher risk for developing AT in an age and sex-dependent manner [179]. Whereas, in the ACL model, overexpression of *VEGFA* may reduce the biomechanical strength of the tendon graft in the early stages of an ACL ligament reconstruction, whilst in the later stages of graft incorporation increased expression is essential [180].

Therefore, it does seem, that the *“Goldilocks affect”* is still plausible [115] suggesting that a finely tuned homeostatic feedback regulation of ECM components is required to maintain both tendon and ligament tissue integrity. Willard and Suijkerbuijk have shown functional evidence linking a genetic contribution, at key proteoglycan, interleukin, and collagen genes, to the expression of ECM components in a susceptibility model [158,164].

Further exploring the angiogenesis pathway and knowing the VEGF biological effects are mediated via its receptor kinase insert-domain receptor (KDR), Rahim showed that the inferred G-A *KDR* haplotype (rs2071559 A/G, rs1870377 A/T) was significantly associated with increased susceptibility to ACL ruptures [174]. It was suggested that the rs2071559 G allele alters a potential transcription factor binding site in the promoter region, thereby reducing *KDR* transcription [181] and the A allele of rs1870377 T/A was associated with reduced VEGF-binding efficiency [181]. Lulinska-Kuklik identified an association between *VEGFA* rs2010963 and ACL injuries in a Polish population, but not with *VEGFA* rs699947 or *VEGFA* rs1570360 [178]. These associations need to be repeated in larger ACL data sets. More recently, Feldmann investigated ACL injury risk in a combined cohort from Sweden, Poland, Australia, and South Africa, and further implicated the *VEGFA* rs201093 CC genotype and the *VEGFA* (rs699947 C/A, rs57036 G/A and rs2010963 G/C) A-A-G haplotype to be associated with reduced risk of an ACL injury [182]. In addition, the authors suggest that possibly variants in *KDR* are not associated with ACL risk susceptibility. The differences noted with *KDR* in the various populations may represent Type I statistical errors (Table 14).

More informative population specific genetic variants are required to be tested [183,184,185,186]. Observing the differences and similarities between these genetic association findings with tendon and ligament injury susceptibility may highlight different biological mechanisms underpinning the two injury models (acute vs. chronic) or it may be indicative of the differences between ligament and tendon molecular-functional correlations. Moreover, it may also be reflective that larger data sets are required to comprehensively screen a gene of interest across populations. It remains essential that the genetic associations are explored at a functional level to assess the biological significance of these variants in both acute and chronic injuries.

## 7. Epigenetics

Despite the growing number of genetic loci implicated in tendon and ligament injury susceptibility, a large unknown heritability component remains unresolved [24]. The impact of epigenetic regulation of the genome is an emerging area of research in understanding and deciphering the heritability of common complex phenotypes. Epigenetics was first described in 1968 [187]. It refers to heritable changes (from parent to child) which do not depend upon DNA sequence changes. DNA within every one of our cells is identical in content and sequencing with a few exceptions. What determines an individual cell’s function is the gene expression within it. Differential expression will determine, for example, if a cell acts as a tenocyte or osteoblast.

DNA must be accessible for transcription by RNA polymerase to be active. Genetic material transcription ‘visibility’ can be controlled by several mechanisms, which are important during development and differentiation of cellular components and tissues. Equally, they are important in influencing the way that mature tendons and ligaments respond to normal adaptation to loading and pathological disease evolution.

The main epigenetic mechanisms facilitating protein expression regulation, without altering the DNA sequence, involve chemical modifications to the genome and DNA, and the associated proteins. This occurs through either DNA methylation, histone modifications and the actions of non-coding RNAs (ncRNA). The latter is an RNA molecule produced by DNA, but not translated into protein. They regulate gene expression at both the transcriptional and post-transcriptional stages of protein synthesis. This type of RNA includes micro-RNAs. They influence and act alongside other epigenetic mechanisms to create ‘gene silencing’. Parts of epigenetic regulation can be inherited via the germline. Others result from a response to an individual’s environmental exposure, which can include but is not limited to lifestyle choices. Epigenetic mechanisms may play an important part in explaining the variation of phenotype in complex disease processes. This includes the lack of reproducibility in some linkage and association studies, observed twin discordance, and different ages of disease-onset when subjects possess the same genotype [188].

### 7.1. DNA Methylation

Some of the genes/gene families implicated in either tendon or ligament injury risk susceptibility have been associated with altered methylation status [189]. For example, a hypermethylation status, typically associated with reduced gene expression, of several CpG sites within the promoter regions of the *MMP11* and *ADAMTS4* genes but not *TIMP2* was noted in pathological human patellar tendon samples compared to control tendon tissue samples [190,191]. The MMP11 and ADAMTS4 enzymes play an important role in ECM regulation and in particular PGs such as aggrecan. Enzyme reduction could result in substrate accumulation. An increased expression profile of large proteoglycans, such as aggrecan, was reported in pathological human patellar tendons [192,193].

The methylation status, together with the corresponding mRNA expression profiles of several MMPs (*MMP1*, −2, −3, −9, −13, and −14) and TIMP (*TIMP1*, 2 and 3) genes, was investigated using ruptured shoulder supraspinatus tendon tissue [194]. Significantly altered DNA methylation patterns for *MMP1*, *MMP9*, *MMP13*, *TIMP2*, and *TIMP3* were described at the torn tendon edge compared to uninjured control tendon samples. An inverse correlation was noted between the overall methylation status of promotor sites within *MMP1*, −9, −13, and TIMP3, including the 5’-UTR of TIMP3, and the respective mRNA expression. Changes in the methylation pattern of some of these genes were partly influenced by age at surgery, sex, smoking habit, tear size, and/or duration of symptoms, which are some of the confounders previously implicated in injury susceptibility and/or impaired tendon healing [158].

### 7.2. RNA Interference

There has been increased interest in miRNAs and miRNA binding sites and their role in protein expression regulation at the post-translational level. In particular, the association of the putative hsa-miR-608 binding site within the 3’UTR region of *COL5A1* gene with both AT and ACL rupture susceptibility [54,58].

The miRNA-29 family, for which direct/indirect targets include collagens, MMPs, integrins and some DNA methylases have been examined [195]. No significant differences in expression profile were noted in torn supraspinatus tendons versus uninjured tendon samples. However, there was an inverse correlation between the expression profiles of hsa-miR-29a-3p, hsa-miR-29b-3p and -5p and *MMP2*, *MMP9* and *MMP14* expression. hsa-miR-29a-3p and miR-29b-5p were inversely correlated with MMP1 expression [194]. This suggests that the miRNA-29 family contributes to regulating specific ECM components of rotator cuff tendons. 

Several potential epigenetic candidate loci and regulators have been highlighted in both tendon and ligament biology. However, little has been explored in the context of tendon and ligament injury and susceptibility [196].

## 8. Discussion

This review has highlighted both overlaps and distinctions in the genetic loci implicated between tendon and ligament injuries, as well as for acute and chronic injuries of both. This underlines the subtleties within musculoskeletal soft tissue pathology. Underlying factors behind acute, subacute, and chronic pathology are likely to be very different. For acute injuries, extrinsic factors will probably dominate—irrespective of the genotype of the individual involved, e.g., an impact injury. More chronic problems (and those who fail to recover from acute injuries) are more likely to identify intrinsic factors, including genetic, as part of the underlying disease pathway. 

It is important for researchers to avoid categorising all tendon and ligament issues as having a common pathology. Structural changes can affect different parts of the tendon and ligament. For example, the Achilles tendon can have problems arising from within both the main tendon and its calcaneal insertion. The anatomy, physiology, and biomechanics will vary according to site. Within the main tendon, changes can affect the paratenon or main tendon. The medially placed plantaris tendon can be the primary source of ‘Achilles’ pathology’. The tendon may suffer small splits and partial tears or, more commonly, tendon enlargement. An acute rupture needs to be categorised differently to a decade old swollen tendon. These different subsets of potential pathology highlight the importance of the care required when assembling cohorts for genetic research analysis.

It is becoming evident that tendon and ligament matrix remodelling is complex and, therefore, not surprising that genetic loci controlling both structural pathways and regulators of ECM homeostasis have been implicated in injury susceptibility. There appears to be a lag in progression in understanding the functional significance of most of these loci. Preliminary functional theories have been presented for *COL1A1*, *COL5A1*, and for the *VEGFA* loci. It is critical that (i) genetic loci are explored in large data sets, (ii) explored in multiple populations, (iii) and that the functional significances of these loci are explored at the cellular and tissue levels for us to (iv) start determining the clinical relevance of these genetic contributions to normal adaptation and injury susceptibility, recovery, and tissue capacity.

One reason for a lag in expression and functional analyses, is access to pathological tissue to explore gene-phenotype correlations and impact on these tissues of time on the “remodelling curve”. Tendons and ligaments are dynamic tissues capable of responding to changing environments and the capacity to be influenced by both environmental, genetic and non-genetic factors supporting and guiding ECM remodelling. The contributions to date of genetic research on tendon and ligament injury and disease have represented no more than the identification of small pieces in a huge jigsaw puzzle. We are still to fully understand the exact position, importance, and relationships of these pieces.

Most research to date has followed a case-control genetic association approach and have been relatively restricted in location and subject and control ethnicity. The need for larger, more highly powered studies is essential. The ability to replicate the findings of the early studies in more geographical locations and representing more diverse ethnic groupings is essential. The era of omics and high throughput technologies is well-established and there is a growing trend for its application in tendon and ligament injury susceptibility. These approaches are highlighting a few of the susceptibility loci identified in the case-control studies such as *TNC* and *COL5A1*. However, the large majority of these have not been identified in GWAS, whole genome sequencing (WGS), and WES analyses. 

Poorly defined cases and controls have been a limitation of the hypothesis-free approaches to date. Moving forward, cases and controls recruited to genetic studies should be classified as carefully as possible based on the most appropriate clinical and imaging assessment tools. Exposure to other confounding risk factors, an injury profile of the full spectrum of tendon and ligament or any other connective tissue injuries, medication history, pain symptoms, flexibility and other relative measurements need careful documentation. Failure to do so will reduce the probability of identifying important genetic factors at various stages of the underlying pathology, as well as improving our understanding of the capacity of the tissues to resist, respond, and recover from load.

Future work should attempt to determine the relative contribution of different genetic factors on overall risk and the potential influence of epigenetic factors affecting gene expression. Once we have more puzzle pieces fitting, we should be in an improved position towards a golden opportunity of improving patients’ care, towards reducing and preventing musculoskeletal conditions [197]. The ‘holy grail’ of genetic research in this field is a better understanding and management of soft-tissue pathology utilising laboratory-based findings during clinical work. This may arise as part of a preventative programme recognising certain individuals increased susceptibility towards developing certain disorders. Equally, it might identify which patients should respond better to different treatment strategies or improve understanding of why certain management avenues have failed.

The ethical considerations of using such data in a clinical or sporting context must be considered. Within clinical professions, understanding the subtleties of such research results is more readily appreciated when interpreted in a medical context. However, the same does not necessarily apply within a sporting context—particularly in elite sport. Ideally, polygenic profiling of identified gene candidates and scientifically assigned weighting to their importance would allow athletes to be scored to determine relative risk of developing tendon or ligament injury. Preventative programmes could be instituted to minimise risk. However, at this stage, such profiling only identifies relative risk. It carries no certainty of freedom from, or the development of, certain problems. Ethical issues will occur if athletes are selected for, or more importantly denied, sporting opportunities based on such research. Already companies exist globally providing genetic profiling and major sports organisations have screened their athletes [198]. Our level of understanding as clinicians and biologists has not yet reached the point where such developments should be condoned.

## 9. Conclusions

The understanding of tendon and ligament structure, function and pathology and potential genetic influences has increased dramatically in the last decade. This review highlights overlaps and distinctions in the genetic loci implicated in tendon and ligament injuries. It also highlights the need for improved study designs, including well phenotyped participants, larger samples sets, increased utilisation of next generation sequencing technologies, and functional studies of implicated loci towards improved understanding of the molecular mechanisms of these genetic loci in injury biology and their clinical significance. This would facilitate steps towards improved management of the large numbers of problems presenting in health care settings.

Our present knowledge levels are still imprecise and subject to contradictions as new emerging research and technologies appear. Consequently, our ability to translate this information into meaningful patient interventions remains limited. However, the authors are confident that, with the present level of advances in research, the ability to fuse scientific work and clinical applications will emerge in the short- to medium-term.

## Figures and Tables

**Table 1:** Principal tendon collagen types.

Collagen Type	Functions	Associated Diseases
Type I	Major tendon in collagen—90% Primary structural elements	Rare mutations within *COL1A1* and *COL1A2* genes, which encode for the α1 and α2 chains of type I collagen, respectively, cause osteogenesis imperfecta (OGI) [17]
Type III	Regulates type I collagen size (fibrillogenesis) [37] Increases in ageing tendons [38] Important role in flexibility and tissue strength Active in early wound healing and, later, gradually replaced by type I collagen as the wound matures	*COL3A1* mutations have been found to cause vascular type of Ehlers–Danlos syndrome (EDS) [39]
Type V	Similar to type III Regulates fibril assembly and diameter and, thus, affects tendon mechanical properties [40] Content increases with age and in degenerative conditions [41]	Common cause of EDS are mutations that inactivates one copy of *COL5A1* (known as a haploinsufficiency) [39]
Type XI	Regulates type I and type II collagen fibrillogenesis by maintaining fibril spacing and diameter Although predominately expressed together with type II collagen, type XI collagen shares structural and functional homology with type V collagen and expressed in developing tendons [42] Proposed that genetic variants controlling type XI and V collagen production interact to regulate type I collagen fibril assembly [43]	Mutations in type XI collagen genes (*COL11A1* and *COL11A2*) associated with Stickler syndrome, Marshall syndrome, fibrochondrogenesis, otospondylomegaepiphyseal dysplasia deafness, and Weissenbacher–Zweymüller syndrome [44]
Types XII and XIV	Type XII and XIV collagens are associated with type I collagen fibrils forming a molecular bridge between structural collagen and other ECM molecules [45] Type XIV assists collagen fibrillogenesis regulation	Microdeletions on long arm of chromosome 6 containing *COL12A1* gene cause developmental delay, mild dysmorphism and connective tissue laxity [46]

**Table 8:** Tendon disease: Inflammatory cascade, apoptosis, and other elements.

Type	Elements or Activity	Functions	Key Elements
Inflammatory Cascade and Apoptosis	Interleukins (ILs)	Intimately involved in the inflammatory pathway	Interleukin-1ß (IL1B) influences cellular death, proliferation, and differentiation Interleukin-1 receptor antagonist (ILRN) inhibits activities of both IL1B and interleukin-1α (IL1A) Interleukin-6 (IL6) is found in both acute and chronic inflammation
	Caspases (CASP)	Family of protease enzymes intimately involved in apoptosis	CASP8 is a pro-enzyme central to cell death regulation
	Nitric Oxide Synthases (NOS)	Nitric oxide (NO), produced by NOS enzymes, is a free radical and important cellular signalling messenger with many functions including increased production of IL6 and Il8 [107]	Three *NOS* genes, *NOS1*, *NOS2*, and *NOS3*, encode for nNOS, iNOS and eNOS enzymes, respectively These genes are designed to code for differing elements of the inflammatory cascade and control over cell death Gene variations associated with alterations in susceptibility to tendinopathy [108] *NOS2* expression elevated 23-fold higher than controls within days of Achilles’ tendon injury NO catalysed by the iNOS isoform of NOS induces apoptosis in inflammatory cells to eradicate cells from damaged area, preventing chronic inflammation and allowing remodelling to occur [109]
	Angiogenesis	Marked increase in new blood vessels in chronic tendon disease [110]	Vascular endothelial growth factor A (VEGFA) Kinase domain receptor (KDR) Hypoxia inducible factor 1 subunit alpha (HIFIA) Encoded by *VEGFA, KDR*, and *HIF1A* genes, respectively [111,112,113]
Others	Estrogen-related receptor beta (ERR-β)	Function unknown May influence the expression of PPARGC1 and ESRR-inducing regulator muscle 1 (PERM1) in skeletal muscle	
	Defensins	Microbicidal and cytotoxic peptides made by neutrophils	

**Table 10:** Summary of genetic research and ligament injuries: Collagen types I and III.

Collagen Types	Reference	Country	Variant	Pathology	Subjects	Controls	Comments
Type I	[150]	South Africa	*COL1A1* rs1800012	ACL rupture	117	130	Significant association
	[147]	Poland	*COL1A1* rs1107946, rs1800012	ACL rupture	91	143	Significant association
	[146]	Poland	*COL1A1* rs1107946, rs1800012	ACL rupture	138	183	Significant association
	[142]	Meta analyses	*COL1A1* rs1800012	Tendon and ligament injuries	933	1381	Significant association
	[149]	Chinese Yunnan Han	*COL1A1* rs1800012	ACL rupture	101	110	No significant association
	[50]	Combined population from SA, Sweden, Poland, Finland	*COL1A1* rs1800012	ACL rupture and Cruciate ligament rupture	1425	407	Significant association
Type III	[151]	Poland	*COL3A1* rs1800255	ACL rupture	138	183	Significant association
	[152]	South Africa, Poland	*COL3A1* rs1800255	ACL rupture	333	378	Significant association

Green indicates an association and red indicates no association found.

**Table 11:** Summary of genetic research and ligament injuries: Collagen types V and XII.

Collagen Types	Reference	Country	Variant	Pathology	Subjects	Controls	Comments
Type V	[153]	South Africa	*COL5A1* rs12722, rs13946	ACL rupture	129	216	Significant association
	[157]	Poland	*COL5A1* rs12722, rs13946	ACL rupture	138	183	Associated with reduced injury risk
	[152]	South Africa and Poland	*COL5A1* rs12722	ACL rupture	333	378	Significant gene-gene association
	[154]	Poland	*COL5A1* rs12722, rs13946	ACL rupture	134	211	Significant association
	[158]	South Africa and Sweden	*COL5A1* rs12722	ACL rupture	98 79	116	Significant association
	[156]	South Africa	*COL5A1* rs3922912, rs4841926	ACL rupture	249	210	No significant association
	[149]	Chinese Yunnan Han	*COL5A1* rs12722, rs13946	ACL rupture	101	110	No significant association
	[155]	South Africa, Australia, Japan	*COL5A1* rs12722, rs10628678	ACL rupture and Ligament injury	311	592	Significant association
Type XII	[159]	South Africa	*COL12A1* rs970547	ACL rupture	129	216	Significant association
	[160]	Poland	COL12A1 rs970547	ACL rupture	91	143	No significant association
	[152]	South Africa and Poland	COL12A1 rs970547	ACL rupture	333	378	Significant association
	[149]	Chinese Yunnan Han	*COL12A1* rs970547, rs240736	ACL rupture	101	110	Significant association

Green indicates an association and red indicates no association found.

**Table 12:** Summary of genetic research and ligament injuries: Glycoproteins in the ECM and proteoglycans.

Type	Protein	Reference	Country	Variant	Pathology	Subjects	Controls	Comments
Glycoproteins in the ECM	Tenascin C (TNC)	[30]	South Africa	TNC rs1061494, rs1138545, rs2104772, rs1061495	ACL rupture	234	232	Significant association
		[162]	Poland	TNC rs1330363, rs2104772, rs13321	ACL rupture	229	192	No significant association
Proteoglycans	Aggrecan (ACAN)	[163]	South Africa	*ACAN* rs2351491, rs1042631, rs1516797	ACL rupture	227	234	Significant association
		[165]	Poland	*ACAN* rs2351491	ACL rupture	143	229	Significant association
	Biglycan (BGN)	[163]	South Africa	*BGN* rs11264797, rs1126499, rs1042103	ACL rupture	227	234	Significant association
		[165]	Poland	*BGN* rs11264797, rs1042103	ACL rupture	143	229	Significant association
		[166]	South Africa	*BGN* rs11264797, rs1042103	ACL rupture	227	234	Significant gene-gene interactions
	Decorin (DCN) Lumican (LUM) Fibromodulin (FMOD)	[163]	South Africa	*DCN* rs13312816, rs516115 *LUM* rs2268578 FMOD rs7543148	ACL rupture	227	234	Significant association

Green indicates an association and red indicates no association found.

**Table 14:** Summary of genetic research and tendon injuries: Angiogenesis and fibrinogens.

Type	Protein	Reference	Country	Variant	Pathology	Subjects	Controls	Comments
Angiogenic Factors	Vascular endothelial growth factor receptor (KDR) Vascular endothelial growth factor (VEGF) Beta-nerve growth factor (NGF) Hypoxia-inducible factor (HIF)	[174]	South Africa	*KDR* rs2071559, rs1870377 *VEGFA* rs699947, rs1570360, rs2010963 *NGFB* rs6678788 *HIF1A* rs11549465	ACL rupture	227	126	Significant association
		[177]	South Africa	*KDR* rs2071559, rs1870377 *VEGFA* rs699947, rs1570360, rs2010963	ACL rupture	98	100	Significant association
		[176]	South Africa	*KDR* rs2071559, rs1870377 *VEGFA* rs699947, rs1570360, rs2010963	ACL rupture	234	232	Significant association
		[178]	Poland	*VEGFA* rs699947, rs1570360, rs2010963	ACL rupture	222	190	Significant association for rs2010963
		[182]	Sweden, Poland, Australia and South Africa	*VEGFA* rs699947, rs1570360, rs2010963 *KDR* rs2071559, rs1870377	ACL rupture	912	765	Significant association for rs201093 and the rs699947, rs57036 rs2010963 haplotype
Fibrinogens	Fibrinogen beta chain (FGB)	[149]	Chinese Yunnan Han	FGB rs1800789, rs1800790, rs1800791, rs2227389	ACL rupture	101	110	No significant association

Green indicates an association and red indicates no association found.

## Data Availability

Not applicable.
